# Supplementary material for: Coral photosymbiosis on Mid-Devonian reefs
Source: Nature. 2024 Oct 23;636(8043):647–53. doi: 10.1038/s41586-024-08101-9 (PMC11655356; doi:10.1038/s41586-024-08101-9)
Supplement: Supplementary file 1 — This Supplementary Information includes three supplementary figures for Palaeozoic coral identification and preservation status. It includes five supplementary tables with newly derived CAI values from the Hagen-Balve Reef in Sauerland as well as the 15N, 18O and 13C isotope values for all Palaeozoic and modern corals, with the number of measurements for each species and material. [file 41586_2024_8101_MOESM1_ESM.pdf]

nature portfolio

https://doi.org/10.1038/s41586-024-08101-9

Supplementary information

Coral photosymbiosis on Mid-Devonian reefs

In the format provided by the authors and unedited

Supplementary Information for:

Coral Photosymbiosis on Mid-Devonian Reefs

Jonathan Jung[1]*, Simon F. Zoppe[2], Till Söte[3], Simone Moretti[1], Nicolas N. Duprey[1], Alan D. Foreman[1], Tanja Wald[1], Hubert Vonhof[1], Gerald H. Haug[1], Daniel M. Sigman[4], Andreas Mulch[2,5], Eberhard Schindler[6], Dorte Janussen[6] and Alfredo Martínez-García[1]*

[1] Climate Geochemistry Department, Max Planck Institute for Chemistry, Mainz, Germany

[2] Goethe University Frankfurt, Institute of Geosciences, Frankfurt am Main, Germany

[3] Department of Geology and Paleontology, University of Münster, Münster, Germany

[4] Department of Geosciences, Princeton University, Princeton, New Jersey, United States

[5]Senckenberg Biodiversity and Climate Research Centre, Frankfurt am Main, Germany

[6]Senckenberg Research Institute and Natural History Museum Frankfurt, Frankfurt am Main, Germany

*Corresponding authors emails: jonathan.jung@mpic.de and a.martinez-garcia@mpic.de

Supplementary Information

This supplementary information includes 3 supplementary figures for Paleozoic coral identification and preservation status. Additionally, it includes 5 supplementary tables with newly derived conodont alteration index values from the Hagen-Balve Reef in Sauerland and the nitrogen, oxygen, and carbon isotope values for all Paleozoic and modern corals with the number of measurements for each species and material.

Supplementary Figures

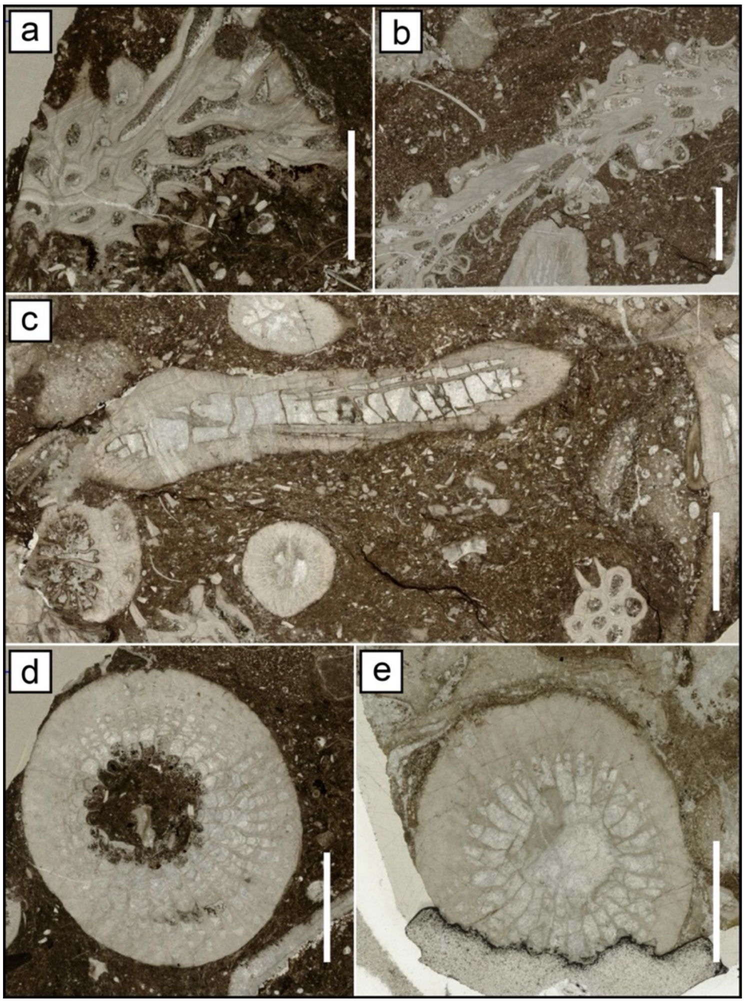

Supplementary Figure 1. Thin sections of Paleozoic material from Sauerland. Microphotographs of thin sections (under transmitted light) of corals from the initial Hagen Balve Reef (lower/middle Givetian) from the Binolen locality in the Sauerland. **a)** Anastomosing branch of tabulate (auloporid) coral *Roemerolites brevis rhiphaeus* (GMM B2C.59-2). **b)** Branch of *R. brevis rhiphaeus* with calices partly filled with sediment and/or sparite (GMM B2C.59-3). **c)** Longitudinal and cross-sections of fasciculate (dendroid) rugose coral *Dendrostella trigemme* and fragments of *R. brevis rhiphaeus* (GMM B2C.59-4). One cross-section of *D. trigemme* (lower left) shows species-specific dendroid astogeny. **d)** Cross-section through calyx of the solitary rugose coral *Temnophyllum astrictum* with species-specific incompletely developed peripheral wall (same thin section as 2a). Calyx center is filled with sediment, whereas spaces between septa and dissepiments are filled with sparite. **e)** Cross section through the solitary rugose coral *Temnophyllum latum* with calyx filled with sparite (GMM B2C.59-9). In contrast to *T. astrictum*, the thick peripheral wall of *T. latum* is nearly completely developed. All thin sections belong to a microfacies type described by Löw et al. (2022) as coral-stromatoporoid baffle-floatstones (their MF8 including subtypes), representing deposits of an initial reef platform of the Hagen-Balve Reef. Scale bars: 5 mm.

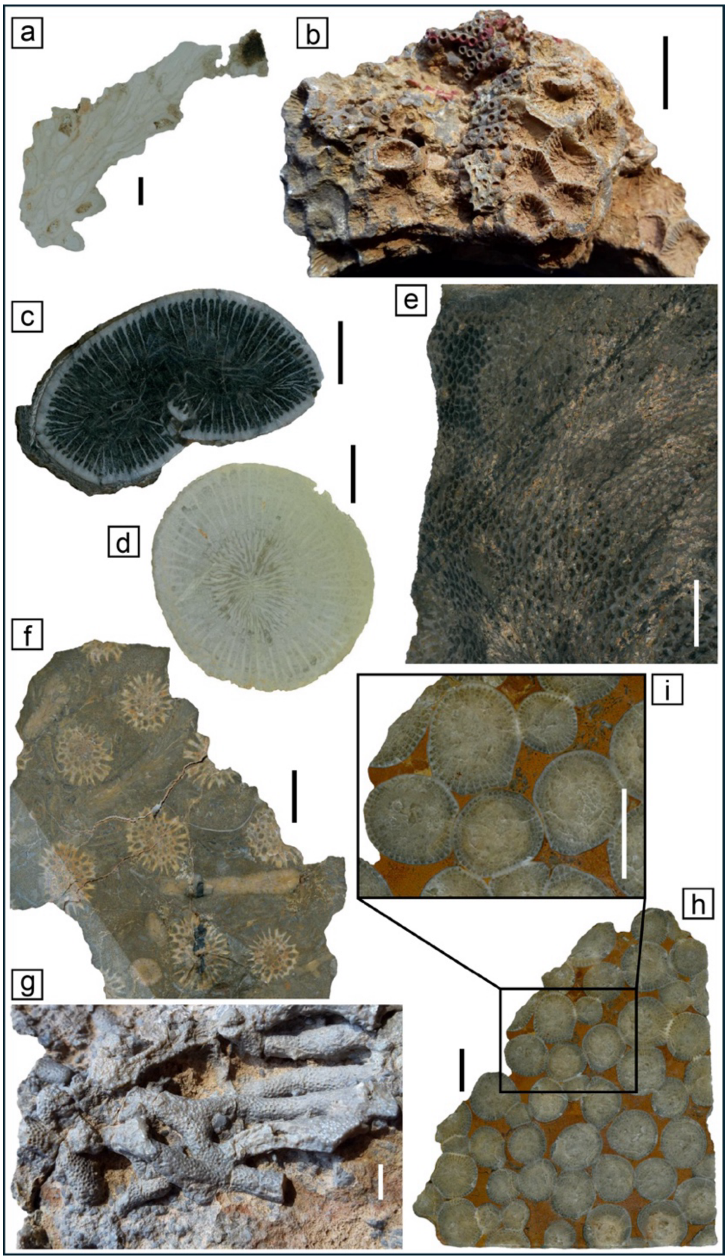

Supplementary Figure 2. Thin sections and photographs of Paleozoic corals from the Eifel, Morocco, and Western Sahara. Coral samples from the collection of the Senckenberg Research Institute and Natural History Museum from the Blankenheim Syncline, Eifel (a-b), Tafilalt, Morocco (c-e), and Sabkhat Lafayrina, Western Sahara (f-i). a) Microphotograph of thin section (under transmitted light) from tabulate (auloporid) coral *Roemerolites brevis brevis* (SMF40160). b) Surface view of the colonial (cerioid) rugose coral *Argutastraea quadrigemina* being partly overgrown by *R. brevis brevis* (SMF40160). c) Cut surface (wetted with water) of the calyx (cross section) of the solitary rugose coral *Siphonophrentis* sp. (SMF75855). d) Microphotograph of thin section (under transmitted light) with the calyx (cross section) of the solitary rugose coral *Acanthophyllum concavum* (SMF75854). e) Polished rock slab with tabulate (alveolitid) coral *Alveolites intermixtus intermixtus* (SMF75856). f) Cut surface of limestone with cross sections of the tabulate (pachyporid) coral *Thamnopora angusta* (Lok.26). g) Surface view of thin branches of the tabulate (alveolitid) coral ?*Scoliopora* sp. (Lok.26). h) Cut surface (wetted with water) with the cross sections of calcices of the fasciculate (phaceloid) rugose coral ?*Dispyllum* sp. (Lok.26). i) Detail of h). Scale bars: 2 mm (a), 10 mm (b, c, e, f, h, i), 5 mm (d, g).

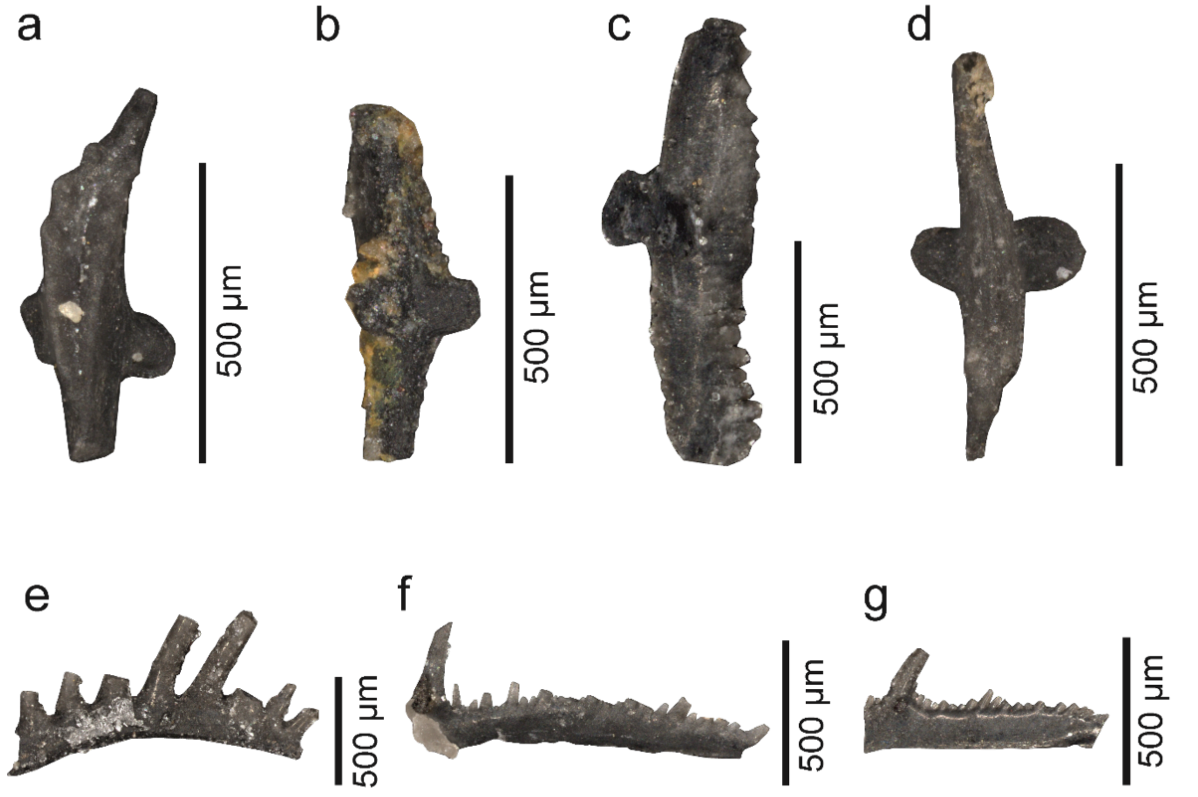

Supplementary Figure 3. Photographs of Conodont fragments from Sauerland. Conodont fragments from Binolen. (a) *Bipennatus bipennatus* MT α, Bed 4, GMM B9A.15-1. (b) *Bi. bipennatus* MT α, Bed 4, GMM B9A.15-2. (c) *Bi. bipennatus* MT α, Bed 18a, GMM B9A.15-15. (d) *Bi. bipennatus* MT α, Bed 29, GMM B9A.15-31. (e) Polygnathid Pb element, Bed 18a, GMM B9A.15-17. (f) Polygnathid ramiform element, Bed 23, GMM B9A.15-26. (g) Polygnathid ramiform element, Bed 23, GMM B9A.15-27.

Supplementary Tables

**Species**	**Bed No. (Löw et al. 2022)**	**GMM No. (Löw et al. 2022)**	**CAI**
Bipennatus bipennatus, MT α	4	GMM B9A.15-1	4
Bi. bipennatus, MT α	4	GMM B9A.15-2	4
Icriodus ?latecarinatus	4	GMM B9A.15-3	4
Drepanodus sp.	4	GMM B9A.15-4	4
?*Neopanderodus* sp.	5	GMM B9A.15-5	4
Prioniodina sp.	6	GMM B9A.15-6	4
Dvorakia chattertoni	6	GMM B9A.15-7	4
Dv. chattertoni	6	GMM B9A.15-8	4
Dv. chattertoni	6	GMM B9A.15-9	4
Belodella resima	6	GMM B9A.15-10	4
Neop. perlineatus	6	GMM B9A.15-11	4
Prioniodina sp.	9	GMM B9A.15-12	4
Panderodus sp.	16	GMM B9A.15-13	4
Bel. resima	16	GMM B9A.15-14	4
Bi. bipennatus, MT α	18a	GMM B9A.15-15	4
Bi. bipennatus, MT α	18a	GMM B9A.15-16	4
Polygnathid Pb element	18a	GMM B9A.15-17	4
?Prioniodid ramiform element	18a	GMM B9A.15-18	4
Bi. bipennatus, MT α	18b	GMM B9A.15-19	4
Bel. resima	20 top	GMM B9A.15-20	4
Dv. chattertoni	20 top	GMM B9A.15-21	4
Polygnathid ramiform element	20 top	GMM B9A.15-22	4
Panderodus cf. *unicostatus*	21	GMM B9A.15-23	4
?*Neopanderodus* sp.	21	GMM B9A.15-24	4
Dv. chattertoni	22	GMM B9A.15-25	4

Polygnathid ramiform element	23	GMM B9A.15-26	4
Polygnathid ramiform element	23	GMM B9A.15-27	4
Bi. bipennatus, MT α	23	GMM B9A.15-28	4
Dv. chattertoni	25	GMM B9A.15-29	4
Bi. bipennatus, MT α	28	GMM B9A.15-30	4
Bi. bipennatus, MT α	29	GMM B9A.15-31	4
Dv. klapperi	50	GMM B9A.15-32	4
Dv. klapperi	50	GMM B9A.15-33	4

Supplementary Table 1. Conodonts from Binolen and their respective CAI value (determination of conodonts by Z. S. Aboussalam and R. T. Becker).

Sample	$\delta^{15}N$	±1SD	nmol N/mg	±1SD	n=
**Hagen Balve Reef - Binolen (Germany)**					
**Marine limestones [host rock]**	1.57(-3.80)	0.46(6.60)	0.71(3.49)	0.18(3.80)	16(10)
**Marine cements [sparite]**	1.45(3.59)	0.58(4.08)	0.35(1.72)	0.21(1.44)	20(12)
**Tabulata [auloporid morphotype]**					
Roemerolites brevis rhiphaeus (1)	1.18(1.34)	0.23(0.99)	0.40(1.08)	0.02(0.55)	2(3)
Roemerolites brevis rhiphaeus (2)	1.63(0.99)	0.30(0.77)	0.41(0.71)	0.01(0.02)	2(3)
Roemerolites brevis rhiphaeus (3)	1.52(3.67)	0.01(0.99)	0.22(0.74)	0.01(0.22)	2(2)
Roemerolites brevis rhiphaeus (4)	2.45(2.70)	0.07(0.53)	0.53(0.60)	0.26(0.09)	4(2)
**Rugosa [dendroid morphotype]**					
Dendrostella trigemme (1)	0.90(-0.66)	0.12(0.16)	0.11(3.74)	0.00(0.04)	3(2)
Dendrostella trigemme (2)	2.38(1.79)	0.15(1.34)	0.36(0.37)	0.06(0.01)	2(2)
Dendrostella trigemme (3)	0.86(2.42)	0.06(2.16)	0.37(1.75)	0.12(2.46)	3(3)
Dendrostella trigemme (4)	1.47(2.37)	0.43(0.58)	0.41(2.91)	0.13(0.79)	3(2)
Dendrostella trigemme (5)	2.18	0.1	0.3	0.01	2(-)
**Rugosa [solitary morphotypes]**					
Temnophyllum latum	5.16(2.05)	0.88(0.79)	1.06(1.39)	0.64(0.66)	5(3)
Temnophyllum astrictum	5.52(1.60)	1.49(0.45)	0.53(0.55)	0.33(0.27)	4(6)
Rugosa indet.	3.57(1.37)	0.22(1.98)	0.31(1.75)	0.14(1.60)	6(4)
**Eifel Region – Dollendorf Syncline (Germany)**					
**Marine limestone [host rock]**	0.83	0.13	1.47	0.05	4
**Rugosa [solitary morphotype]**					
Mesophyllum (*M.*) *vesiculosum*	3.06	0.47	0.32	0.09	7
**Eifel Region – Sötenich Syncline**					

**Marine limestone [host rock]**	1.27	0.16	1.15	0.09	4
**Tabulata [auloporid morphotype]**					
Romerolites brevis brevis	1.22	0.08	0.32	0.05	7
Romerolites brevis brevis	2.24	0.21	0.38	0.03	3
**Rugosa [solitary morphotype]**					
Temnophyllum latum	4.01	0.42	0.89	0.13	7
**<u>Eifel Region – Blankenheim Syncline (Germany)</u>**					
**Marine limestone [host rock]**	2.18(6.06)	0.31(2.79)	0.99(7.26)	0.11(6.63)	6(6)
**Tabulata [pachyporid and auloporid morphotypes]**					
Thamnopora cervicornis (1)	3.02	0.06	0.65	0.04	3
Thamnopora cervicornis (2)	3.35	0.35	0.21	0.01	3
Thamnopora cervicornis (3)	2.98	0.48	0.56	0.03	3
Roemerolites brevis brevis	2.84	0.18	0.59	0.01	3
Thamnopora urensis	3.66	0.26	0.25	0.09	3
**Rugosa [cerioid morphotype]**					
Argutastraea quadrigemina	5.94	0.40	0.17	0.02	3
**<u>Tafialt Basin (Morocco)</u>**					
**Marine limestone [host rock]**	2.84(1.57)	0.08(2.51)	0.34(1.51)	0.0.04(0.43)	6(6)
**Tabulata [alveolitid morphotype]**					
Alveolites intermixtus intermixtus	3.46	0.49	0.43	0.06	3
Alveolites intermixtus minor	3.06	0.44	0.25	0.02	3
**Rugosa [solitary morphotypes]**					

Mesophyllum cf. (*M.*) *lissingenense*	6.33	0.12	0.50	0.09	3
Siphonophrentis sp.	6.03	0.86	0.31	0.09	3
Acanthophyllum concavum	5.87	0.12	0.33	0.09	3
**<u>Sabkhat Lafayrina (Western Sahara)</u>**					
**Marine limestone [host rock]**	3.64(6.55)	0.14(2.34)	0.54(2.00)	0.23(1.50)	6(6)
**Tabulata [pachyporid and alveolitid morphotypes]**					
Thamnopora angusta	4.13	0.10	0.52	0.05	3
?*Scoliopora* sp.	3.48	0.15	0.30	0.02	3
**Rugosa [phaceloid morphotype]**					
?*Disphyllum* sp.	7.34	0.23	0.30	0.03	3
**Rugosa [solitary morphotypes]**					
Mesophyllum (*C.*) *secundum*	6.78	0.42	0.19	0.02	3
Acanthophyllum concavum	7.69	0.12	0.23	0.00	3

Supplementary Table 2. Raw nitrogen isotope and N content/mg values for clean and unclean samples from the Devonian. As expected, unclean samples contain more N/mg and show a greater variation, which implies that cleaning of carefully extracted sample material is preferential. Note that unclean samples are given in parentheses next to the respective clean value. Binolen samples were collected and provided by Simon F. Zoppe. Samples from the Eifel area, from Morocco, and Western Saharan were provided by the Senckenberg Research Institute and Natural History Museum Frankfurt, Germany.

Sample	$\delta^{15}N$	±1SD	nmol N/mg	±1SD	n=
**Cape Verde [São Tiago]**					
Tubastraea sp.	9.17	0.19	3.85	0.66	7
Siderastrea radians	6.83	0.19	2.4	0.17	3
**Colombia [Magdalera]**					
Tubastraea aurea	8.93	0.17	3.49	0.14	6
Tubastraea tenuilamellosa	8.95	0.46	3.31	0.37	5
Gardineria minor	10.42	0.02	2.78	0.02	3
Porites porites	7.14	0.44	2.16	0.06	8
Porites asteroides	5.69	0.05	1.22	0.01	2
Mycetophyllia ferox	6.05	0.89	3.02	0.24	2
**Jamaica [Collection A. Podzorski]**					
Tubastraea aurea (1)	6.91	0.18	2.72	0.11	4
Tubastraea aurea (2)	8.16	0.25	4.71	0.74	4
Thalamophyllia riisei	6.28	0.58	1.99	0.29	2
Phyllangia americana	7.53	0.17	7.66	0.95	3
Porites porites	2.72	0.18	2.06	0.1	5
Porites furcata	3.01	0.25	2.58	0.01	2
Siderastrea siderea	2.82	0.29	2.25	0.25	5
**Socotra [Gulf of Aden]**					
Tubastraea faulkneri	12.74	0.27	3.93	0.17	4
Tubastraea micanthra (1)	12.54	0.11	5.23	0.81	4
Tubastraea micanthra (2)	12.35	0.27	3.58	0.4	4
Porites harrisoni	10.72	0.39	4.25	0.74	4
Porites lobata (1)	10.87	0.12	3.53	0.4	4
Porites lobata (2)	10.19	0.15	3.37	1.06	4

Supplementary Table 3. Pairs of nitrogen isotope and N content/mg values for clean samples from a range of different locations. Symbiont-barren (i.e., *Tubastraea* spp.) and symbiont-bearing (i.e., *Porites* spp.) species were sampled from the same location based on collections at the Senckenberg Research Institute and Natural History Museum Frankfurt, Germany.

Sample	$\delta^{13}C$	±1SD	$\delta^{18}O$	±1SD	n=
**Hagen Balve Reef - Binolen (Germany)**					
**Marine limestones [host rock]**	2.39	0.04	-6.64	0.18	7
**Marine cements [sparite]**	2.29	0.21	-5.57	0.35	19
**Tabulata [auloporid morphotype]**					
Roemerolites brevis rhiphaeus (1)	1.17	0.05	-5.77	0.01	3
Roemerolites brevis rhiphaeus (2)	1.33	0.4	-6.13	0.33	3
Roemerolites brevis rhiphaeus (3)	2.06	0.42	-6.15	0.25	3
Roemerolites brevis rhiphaeus (4)	2.54	0.01	-5.95	0.1	2
**Rugosa [dendroid morphotype]**					
Dendrostella trigemme (1)	1.29	0.07	-6.32	0.04	2
Dendrostella trigemme (2)	1.42	0.05	-6.15	0.09	2
Dendrostella trigemme (3)	0.91	0.07	-5.69	0.01	3
**Rugosa [solitary morphotypes]**					
Temnophyllum latum	1.93	0.51	-5.38	0.19	8
Temnophyllum astrictum	2.14	0.15	-6.03	0.15	8
Rugosa indet.	2.29	0.17	-5.82	0.1	4
**Eifel Region – Dollendorf Syncline (Germany)**					
**Marine limestone [host rock]**	2.05	0.04	-6.47	0.06	3
**Rugosa [solitary morphotype]**					
Mesophyllum (*M.*) *vesiculosum*	0.74	0.07	-5.29	0.09	3
**Eifel Region – Sötenich Syncline**					
**Marine limestone [host rock]**	2.14	0.1	-6.09	0.11	3

**Tabulata [auloporid morphotype]**					
Romerolites brevis brevis	0.59	0.01	-5.6	0.03	3
**<u>Eifel Region – Blankenheim Syncline (Germany)</u>**					
**Marine limestone [host rock]**	2.87	0.01	-5.08	0.03	3
**Tabulata [pachyporid and auloporid morphotypes]**					
Thamnopora cervicornis (1)	2.15	0.03	-5.47	0.03	3
Thamnopora cervicornis (2)	2.79	0.02	-6.06	0.02	3
Thamnopora cervicornis (3)	2.85	0.06	-6.22	0.06	3
Roemerolites brevis brevis	2.37	0.01	-6.44	0.03	3
Thamnopora urensis	2.24	0.02	-6.62	0.06	3
**Rugosa [cerioid morphotype]**					
Argutastraea quadrigemina	1.75	0.06	-7.4	0.05	3
**<u>Tafialt Basin (Morocco)</u>**					
**Marine limestone [host rock]**	2.58	0.03	-4.74	0.04	3
**Tabulata [alveolitid morphotype]**					
Alveolites intermixtus intermixtus	2.84	0.05	-5.46	0.04	3
Alveolites intermixtus minor	2.64	0.04	-6.17	0.07	3
**Rugosa [solitary morphotypes]**					
Mesophyllum cf. (*M.*) *lissingenense*	2.83	0.02	-5.93	0.04	3
Siphonophrentis sp.	2.7	0.06	-6.6	0.05	3
Acanthophyllum concavum	1.48	0.05	-6.61	0.03	3
**<u>Sabkhat Lafayrina (Western Sahara)</u>**					

**Marine limestone [host rock]**	1.82	0.02	-6.84	0.02	3
**Tabulata [pachyporid and alveolitid morphotypes]**					
Thamnopora angusta	2.01	0.04	-4.42	0.03	3
?*Scoliopora* sp.	1.42	0.02	-5.15	0.02	3
**Rugosa [phaceloid morphotype]**					
?*Disphyllum* sp.	3.36	0.05	-3.98	0.01	3
**Rugosa [solitary morphotypes]**					
Mesophyllum (*C.*) *secundum*	0.67	0,05	-6.07	0.05	3
Acanthophyllum concavum	-0.98	0.05	-6.54	0.02	3

Supplementary Table 4. Carbon and oxygen isotope values for our Paleozoic samples from the Hagen Balve Reef, the Eifel, Tafilalt in Morocco and Sabkhat Lafayrina, Western Sahara. Individual values are given and summarized as means for different morphotypes. Binolen samples were collected and provided by Simon F. Zoppe. Samples from the Eifel area, from Morocco, and Western Saharan were provided by the Senckenberg Research Institute and Natural History Museum Frankfurt, Germany.

Sample	$\delta^{13}C$	±1SD	$\delta^{18}O$	±1SD	n=
**Cape Verde [São Tiago]**					
Tubastraea sp.	-6.63	0.07	-2.77	0.18	4
Siderastrea radians	-1.6	0.1	-3.44	0.03	4
**Colombia [Magdalera]**					
Tubastrea aurea	-10.27	0.29	-4.5	0.13	4
Tubastraea tenuilamellosa	-8.16	0.09	-3.85	0.03	3
Gardineria minor	-2.87	0.2	-1.39	0.11	3
Porites porites	-1.66	0.02	-3.98	0.08	3
Porites asteroides	-1.98	0.06	-4.81	1.82	4
Mycetophyllia ferox	-3.07	1.25	-2.85	0.43	4
**Jamaica [Collection A. Podzorski]**					
Tubastraea aurea (1)	-4.7	0.13	-2.53	0.06	3
Tubastraea aurea (2)	-5.77	1.19	-2.99	0.51	3
Thalamophyllia riisei	1.6	0.35	-0.09	0.53	3
Phyllangia americana	-3.79	1.64	-2.26	0.35	4
Porites porites	-3.45	0.06	-3.59	0.01	5
Porites furcata	-2.06	0.31	-2.75	0.07	2
Siderastrea siderea	-0.75	0.05	-3.4	0.02	3
**Socotra [Gulf of Aden]**					
Tubastraea faulkneri	-6.18	0.18	-3.16	0.06	4
Tubastraea micanthra (1)	-9.01	0.05	-3.85	0.12	3
Tubastraea micanthra (2)	-8.12	0.15	-3.43	0.14	3
Porites harrisoni	-2.21	0.1	-3.91	0.05	4
Porites lobata (1)	-1.95	0.09	-2.89	0.04	3
Porites lobata (2)	-2.13	0.04	-3.24	0.02	3

Supplementary Table 5. Carbon and oxygen isotope values for recent pairs of symbiont-barren and symbiont-bearing species from a range of different locations. Skeletal material was sampled at the Senckenberg Research Institute and Natural History Museum Frankfurt, Germany.
