# Supplementary material for: Coral photosymbiosis on Mid-Devonian reefs
Source: Nature. 2024 Oct 23;636(8043):647–53. doi: 10.1038/s41586-024-08101-9 (PMC11655356; doi:10.1038/s41586-024-08101-9)
Supplement: Supplementary file 2 — Reporting Summary [file 41586_2024_8101_MOESM2_ESM.pdf]

nature portfolio

Corresponding author(s): Jonathan Jung and Alfredo Martinez-Garcia

Last updated by author(s): Jun 4, 2024

# Reporting Summary

Nature Portfolio wishes to improve the reproducibility of the work that we publish. This form provides structure for consistency and transparency in reporting. For further information on Nature Portfolio policies, see our Editorial Policies and the Editorial Policy Checklist.

Please do not complete any field with "not applicable" or n/a. Refer to the help text for what text to use if an item is not relevant to your study.
For final submission: please carefully check your responses for accuracy; you will not be able to make changes later.

## Statistics

For all statistical analyses, confirm that the following items are present in the figure legend, table legend, main text, or Methods section.

| n/a | Confirmed | |
|---|---|---|
| ☐ | ☒ | The exact sample size (*n*) for each experimental group/condition, given as a discrete number and unit of measurement |
| ☐ | ☒ | A statement on whether measurements were taken from distinct samples or whether the same sample was measured repeatedly |
| ☐ | ☒ | The statistical test(s) used AND whether they are one- or two-sided<br>*Only common tests should be described solely by name; describe more complex techniques in the Methods section.* |
| ☒ | ☐ | A description of all covariates tested |
| ☒ | ☐ | A description of any assumptions or corrections, such as tests of normality and adjustment for multiple comparisons |
| ☐ | ☒ | A full description of the statistical parameters including central tendency (e.g. means) or other basic estimates (e.g. regression coefficient) AND variation (e.g. standard deviation) or associated estimates of uncertainty (e.g. confidence intervals) |
| ☐ | ☒ | For null hypothesis testing, the test statistic (e.g. *F*, *t*, *r*) with confidence intervals, effect sizes, degrees of freedom and *P* value noted<br>*Give P values as exact values whenever suitable.* |
| ☒ | ☐ | For Bayesian analysis, information on the choice of priors and Markov chain Monte Carlo settings |
| ☒ | ☐ | For hierarchical and complex designs, identification of the appropriate level for tests and full reporting of outcomes |
| ☒ | ☐ | Estimates of effect sizes (e.g. Cohen's *d*, Pearson's *r*), indicating how they were calculated |

*Our web collection on statistics for biologists contains articles on many of the points above.*

## Software and code

Policy information about availability of computer code

| | |
|---|---|
| Data collection | All analyses were conducted using Python3 on a Jupyter Notebook® (version 5.7.4). Data were imported using the Pandas library and plotted with Seaborn or Matplotlib libraries.<br>The nitrogen isotopes were determined by a purpose-built inlet system coupled to a Thermo MAT253 Plus stable isotope ratio mass spectrometer (software: Isodat version 3.0). Oxygen and carbon isotopes were were measured with an isotope ratio mass spectrometer (IRMS) (Delta V Advantage, Thermo Scientific, Bremen, Germany) which is connected to a GasBench II unit (Thermo Scientific) (software: Isodat version 3.0). |
| Data analysis | No software was used for data analysis and the codes used for figures and data analyses are available on GitHub (https://github.com/marinejon/Coral-Photosymbiosis-on-Mid-Devonian-Reefs.git) |

For manuscripts utilizing custom algorithms or software that are central to the research but not yet described in published literature, software must be made available to editors and reviewers. We strongly encourage code deposition in a community repository (e.g. GitHub). See the Nature Portfolio guidelines for submitting code & software for further information.

## Data

Policy information about availability of data

All manuscripts must include a data availability statement. This statement should provide the following information, where applicable:

- Accession codes, unique identifiers, or web links for publicly available datasets
- A description of any restrictions on data availability
- For clinical datasets or third party data, please ensure that the statement adheres to our policy

All data are available in the Supplementary Tables 2 - 5 and upon publication data will be stored in Pangaea (https://www.pangaea.de/)

## Research involving human participants, their data, or biological material

Policy information about studies with human participants or human data. See also policy information about sex, gender (identity/presentation), and sexual orientation and race, ethnicity and racism.

| | |
|---|---|
| Reporting on sex and gender | N/A |
| Reporting on race, ethnicity, or other socially relevant groupings | N/A |
| Population characteristics | N/A |
| Recruitment | N/A |
| Ethics oversight | N/A |

Note that full information on the approval of the study protocol must also be provided in the manuscript.

# Field-specific reporting

Please select the one below that is the best fit for your research. If you are not sure, read the appropriate sections before making your selection.

☐ Life sciences ☐ Behavioural & social sciences ☒ Ecological, evolutionary & environmental sciences

For a reference copy of the document with all sections, see nature.com/documents/nr-reporting-summary-flat.pdf

# Ecological, evolutionary & environmental sciences study design

All studies must disclose on these points even when the disclosure is negative.

| | |
|---|---|
| Study description | Pairs of modern symbiont-bearing and symbiont-barren samples were analyzed to define the isotopic offset (carbon, oxygen and nitrogen isotopes) between the two ecological groups. Samples of Paleozoic groups and different morphologies were analyzed for carbon, oxygen and nitrogen isotopes and compared to the modern pairs. Carbon and oxygen isotopes were used because of prior studies and to show that the direct comparison between modern and Paleozoic corals does not yield a comprehensive picture to differentiate between ancient symbiont-bearing and -barren species. Nitrogen isotopes analyses were used as a new way to distinguish between ecological groups of the Paleozoic. |
| Research sample | Fossilized coral samples of the groups Tabulata and Rugosa from the Paleozoic taken from the Sauerland and Eifel region in Germany as well as Tafilalt in Morocco and Sabkhat Lafayrina in West Sahara. Furthermore, we analyzed modern scleractinian corals from several locations as indicated in Extended Data Figure 2 and Supplementary Table 3 & 5. |
| Sampling strategy | For the Paleozoic samples, we used specimens that provided sufficient material to compare uncleaned and cleaned material (as indicated in Figure 2 and Extended Data Figure 4). Sample material was drilled with a hand-dremel (0.9 mm drill-bit) and material was only taken from the calcitic phase (as indicated in Extended Data Figure 1). For modern samples, material was drilled from samples that were taken from the same reef location and depth. Coral pieces (~2x2 cm) were cut and crushed in an Achat mortar before being cleaned. |
| Data collection | Nitrogen Isotope data were analyzed at the Stable Isotope Laboratory of the Max-Planck Institute for Chemistry in Mainz, Germany. Data were mainly analyzed by Jonathan Jung with the supervision of Alfredo-Martinez-Garcia. Carbon and Oxygen Isotopes were analyzed in the inorganic stable isotope laboratory at the Max-Planck-Institute for Chemistry in Mainz, Germany. |
| Timing and spatial scale | Isotopic analysis were conducted between October 2019 and December 2023. Paleozoic coral samples were taken from two locations in Germany with different diagenetic histories as well as two locations from the opposite basin of the Rheic Ocean (Morocco and West Sahara) and compared to modern coral samples from a range of underlying environmental conditions from which we expected different nitrogen isotope values for symbiont-bearing corals. Our rational was to show that despite different |

| | |
|---|---|
| | underlying environmental conditions (i.e., oligotrophic vs. eutrophic) the difference between symbiont-bearing and symbiont-barren nitrogen isotope values stays the same. |
| Data exclusions | No samples were excluded. |
| Reproducibility | Samples of the same specimens were analyzed over several batches and reproducibility is given as the standard deviation. |
| Randomization | Samples did not need to be grouped since there are no inter-dependencies between species. |
| Blinding | Samples were analyzed in a random order but had to be indicated by labels. |

Did the study involve field work? ☒ Yes ☐ No

## Field work, collection and transport

| | |
|---|---|
| Field conditions | At Sauerland, hand pieces were collected off the ground. Collection happened in October 2018, while It was cloudy but dry at 13°C. |
| Location | The main material was collected near a cliff at the top of the Binolen section ("C-Layers" after Löw et al., 2022; GPS: 51°22'12''N, 7° 51'27''E) within the Hönne Valley in north-western Sauerland. |
| Access & import/export | Samples were taken close to a publicly accessible forest trail where no permits are needed. Pieces were exclusively collected from the ground. |
| Disturbance | Only those hand-pieces were collected that could be picked up from the ground. |

# Reporting for specific materials, systems and methods

We require information from authors about some types of materials, experimental systems and methods used in many studies. Here, indicate whether each material, system or method listed is relevant to your study. If you are not sure if a list item applies to your research, read the appropriate section before selecting a response.

### Materials & experimental systems

| n/a | Involved in the study |
|---|---|
| ☒ | ☐ Antibodies |
| ☒ | ☐ Eukaryotic cell lines |
| ☐ | ☒ Palaeontology and archaeology |
| ☒ | ☐ Animals and other organisms |
| ☒ | ☐ Clinical data |
| ☒ | ☐ Dual use research of concern |
| ☒ | ☐ Plants |

### Methods

| n/a | Involved in the study |
|---|---|
| ☒ | ☐ ChIP-seq |
| ☒ | ☐ Flow cytometry |
| ☒ | ☐ MRI-based neuroimaging |

## Palaeontology and Archaeology

| | |
|---|---|
| Specimen provenance | Sampling permits for specimens from the the Senckenberg Research Institute and Natural History Museum Frankfurt, Germany were issued. No permits were needed otherwise. |
| Specimen deposition | Thin sections for species identification from the initial Hagen Balve Reef at Binolen will be stored in the Geomuseum of the Westfälische Wilhelms University in Münster (GMM) under the inventory numbers GMM B2C.59-1 to GMM B2C.59-9. Sample material from the Eifel and the modern samples are stored at the Senckenberg Research Institute and Natural History Museum Frankfurt, Germany. All sample aliquots are stored at the Max-Planck Institute for Chemistry in Mainz. |
| Dating methods | No new dates were obtained. |

☐ Tick this box to confirm that the raw and calibrated dates are available in the paper or in Supplementary Information.

| | |
|---|---|
| Ethics oversight | No ethical approval was necessary given that no endangered species were analyzed. |

Note that full information on the approval of the study protocol must also be provided in the manuscript.

## Plants

| | |
|---|---|
| Seed stocks | *Report on the source of all seed stocks or other plant material used. If applicable, state the seed stock centre and catalogue number. If plant specimens were collected from the field, describe the collection location, date and sampling procedures.* |
| Novel plant genotypes | *Describe the methods by which all novel plant genotypes were produced. This includes those generated by transgenic approaches, gene editing, chemical/radiation-based mutagenesis and hybridization. For transgenic lines, describe the transformation method, the number of independent lines analyzed and the generation upon which experiments were performed. For gene-edited lines, describe the editor used, the endogenous sequence targeted for editing, the targeting guide RNA sequence (if applicable) and how the editor was applied.* |
| Authentication | *Describe any authentication procedures for each seed stock used or novel genotype generated. Describe any experiments used to assess the effect of a mutation and, where applicable, how potential secondary effects (e.g. second site T-DNA insertions, mosiacism, off-target gene editing) were examined.* |





This checklist template is licensed under a Creative Commons Attribution 4.0 International License, which permits use, sharing, adaptation, distribution and reproduction in any medium or format, as long as you give appropriate credit to the original author(s) and the source, provide a link to the Creative Commons license, and indicate if changes were made. The images or other third party material in this article are included in the article's Creative Commons license, unless indicated otherwise in a credit line to the material. If material is not included in the article's Creative Commons license and your intended use is not permitted by statutory regulation or exceeds the permitted use, you will need to obtain permission directly from the copyright holder. To view a copy of this license, visit http://creativecommons.org/licenses/by/4.0/
